# Antibiotic-Efficient Genetic Cassette for the TEM-1 β-Lactamase That Improves Plasmid Performance

**DOI:** 10.1021/acssynbio.1c00393

**Published:** 2022-01-04

**Authors:** Alister J. Cumming, Diana Khananisho, Ramona Harris, Carolyn N. Bayer, Morten H. H. Nørholm, Sara Jamshidi, Leopold L. Ilag, Daniel O. Daley

**Affiliations:** † Department of Biochemistry and Biophysics, Stockholm University, Stockholm SE106 91, Sweden; ‡ The Novo Nordisk Foundation Center for Biosustainability, Technical University of Denmark, Kongens Lyngby 2800, Denmark; § CloneOpt AB, Upplands Väsby SE194 68, Sweden; ∥ Mycropt ApS, Kongens Lyngby 2800, Denmark; # Department of Materials and Environmental Chemistry, Stockholm University, Stockholm SE106 91, Sweden

**Keywords:** expression plasmid, genetic cassette, β-lactamase, directed evolution, translation initiation region, antibiotic stewardship

## Abstract

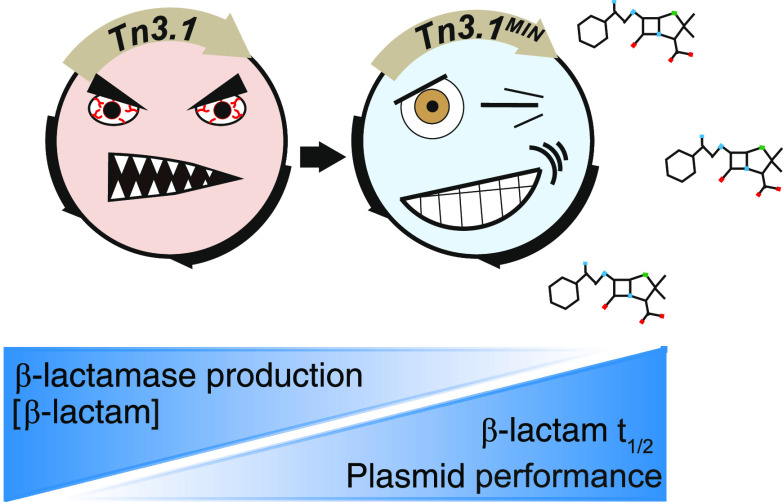

Antibiotic resistance cassettes are indispensable tools in recombinant DNA technology, synthetic biology, and metabolic engineering. The genetic cassette encoding the TEM-1 β-lactamase (denoted Tn3.1) is one of the most commonly used and can be found in more than 120 commercially available bacterial expression plasmids (*e.g.*, the *pET*, *pUC*, *pGEM*, *pQE*, *pGEX*, *pBAD*, and *pSEVA* series). A widely acknowledged problem with the cassette is that it produces excessively high titers of β-lactamase that rapidly degrade β-lactam antibiotics in the culture media, leading to loss of selective pressure, and eventually a large percentage of cells that do not have a plasmid. To address these shortcomings, we have engineered a next-generation version that expresses minimal levels of β-lactamase (denoted Tn3.1^MIN^). We have also engineered a version that is compatible with the Standard European Vector Architecture (SEVA) (denoted Ap (pSEVA#1^MIN^--)). Expression plasmids containing either Tn3.1^MIN^ or Ap (pSEVA#1^MIN^--) can be selected using a 5-fold lower concentration of β-lactam antibiotics and benefit from the increased half-life of the β-lactam antibiotics in the culture medium (3- to 10-fold). Moreover, more cells in the culture retain the plasmid. In summary, we present two antibiotic-efficient genetic cassettes encoding the TEM-1 β-lactamase that reduce antibiotic consumption (an integral part of antibiotic stewardship), reduce production costs, and improve plasmid performance in bacterial cell factories.

## Introduction

**Figure 1:**
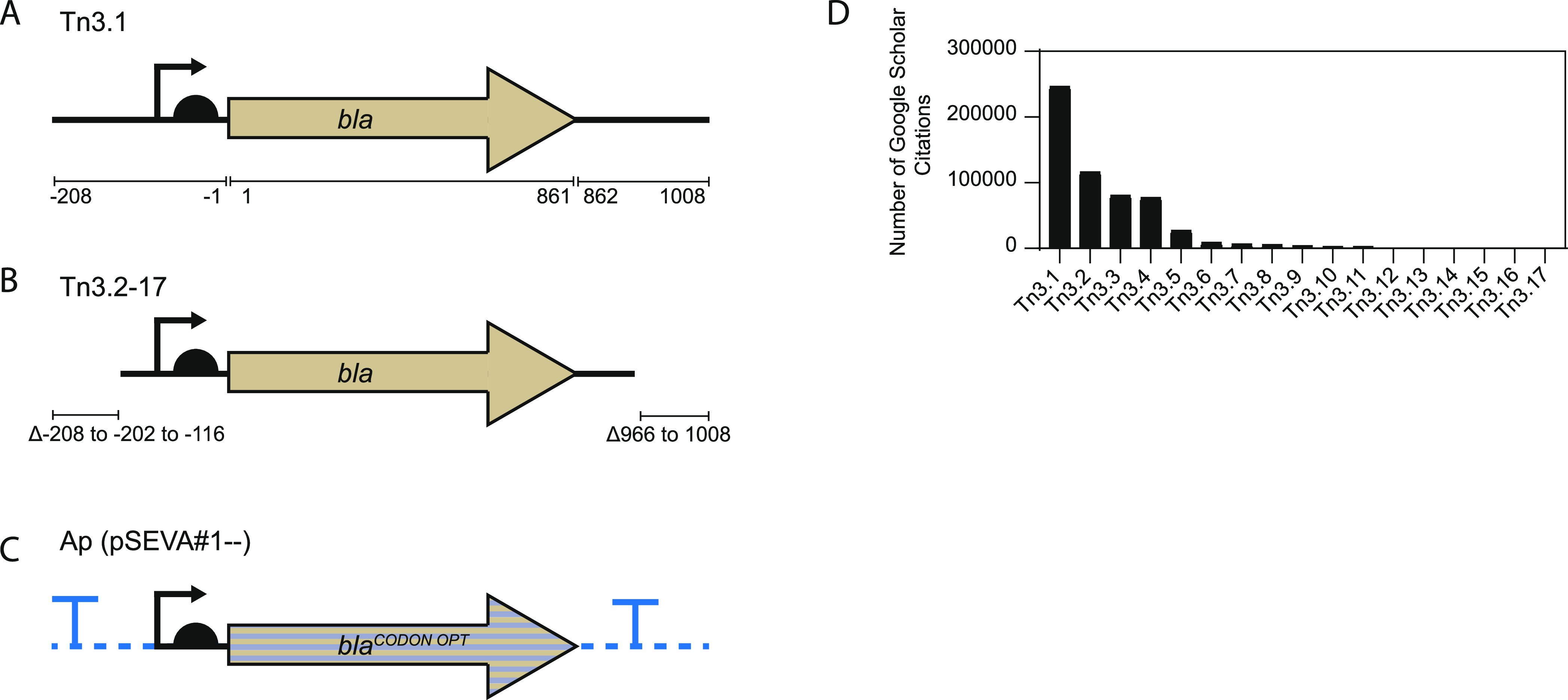
Commonly used genetic cassettes encoding the TEM-1 β-lactamase. (A) Tn3 fragment from the *R1* plasmid of *S. paratyphi* B (herein called Tn3.1) is 1216 nucleotides long. It contains the *bla* coding sequence as well as 5′ and 3′UTRs. The 5′UTR contains a P3 promoter and Shine–Dalgarno sequence. Numbers correspond to the AUG start codon of *bla*. The full sequence of Tn3.1 is shown in Table 1. (B) Versions of the Tn3.1 fragment that are truncated in the 5′ and 3′ UTR are commonly used (herein called Tn3.2–Tn3.17). These versions may also contain nucleotide substitutions (summarized in Table 1). (C) The pSEVA collection contains a codon-varied genetic module encoding the TEM-1 β-lactamase and is flanked by transcriptional terminators and restriction enzyme recognition sites (D). Estimated frequency in the literature of the Tn3-based fragments. Expression plasmids containing the various fragments were searched on Google Scholar, and the number of citations was recorded.

β-Lactamases are a large family of proteins that inactivate β-lactam antibiotics, such as ampicillin and carbenicillin, by enzymatically cleaving the amide bond of the β-lactam ring.^1,2^ The TEM-1 β-lactamase was the first of the family to be discovered in the 1960s.^3,4^ It was encoded on the *R1* plasmid in a wild-type isolate of *Salmonella paratyphi* B and cloned in the process of constructing the pBR322 plasmid (reviewed in ref (5)). Sequencing indicated that a 1216-nucleotide-long fragment from the Tn3 transposon of the *R1* plasmid had been captured (herein referred to as Tn3.1). The Tn3.1 fragment contained the 861-nucleotide-long coding sequence for the TEM-1 β-lactamase (*bla*), as well as 208 nucleotides upstream and 147 nucleotides downstream.^6^ The upstream sequence contained the constitutive *P3* promoter and a Shine–Dalgarno (SD) sequence that was positioned five nucleotides upstream of the *AUG* start codon (Figure 1A).

The Tn3.1-based fragments are widely used as a selection marker in bacterial expression plasmids. For example, 49 commercially available expression plasmids contain the original Tn3.1 fragment (Table 1). Another 84 use versions that are slightly truncated and that contain nucleotide substitutions. One of these nucleotide substitutions deleted a *Pst*I restriction enzyme recognition sequence, but the remainder have not, to our knowledge, been described in the literature. We refer to these altered fragments as Tn3.2–Tn3.17 (Figure 1B and Table 1). A variant of the Tn3.1 fragment has also recently been developed for the Standard European Vector Architecture (*pSEVA*).^7,8^ This version, which is referred to as Ap (pSEVA#1--), contains a codon-optimized version of *bla* that is insulated with transcriptional terminators (from the *trpA* gene of *Escherichia coli* and the *gene VIII* from phage fd) and is flanked by restriction enzyme recognition sites for multifragment assembly (*Swa*I and *Psh*A1) in the SEVA system (Figure 1C). Google Scholar citations indicate that expression plasmids containing the Tn3.1–Tn3.17 fragments have been used in >581 000 studies. The most commonly used being Tn3.1, which has been used in >246 000 published studies (Figure 1D).

**Table 1:** Tn3 Fragments Used in Commercial Expression Plasmids^a^

**fragment**	**length**^**Q**^	**sequence**^**R**^	**citations**^**S**^	**expression plasmids using the fragment**
Tn3.1	1–1216	A	246 266	pBR332, pET1*, pET2*, pET3*, pET4*, pET5*, pET6*, pET7*, pET8*, pET11a-d, pET14b, pET15b, pET16b, pET17b, pET19b, pET-DEST42, pET100/D-TOPO, pET100/D-LacZ, pET101/D-LacZ, pET101/D-TOPO, pET102/D-LacZ, pET102/D-TOPO, pET104-DEST, pET104/GW/LacZ, pET104.1-DEST, pET104.1/D/GW-LacZ, pET151/D-TOPO, pET151/D/LacZ, pET160-DEST, pET160/GW/D-TOPO, pET161/GW-CAT, pET300/NT-GW/Ras Kinase, pET300/NT-DEST, pET301/CT-DEST, pET302/NT-his, pGEX-1 lambda T, pGEX-2T, pGEX-2TK, pGEX-3X, pGEX-4T-1, pGEX-4T-2, pGEX-4T-3, pGEX-5X-1, pGEX-6p-1, pGEX-6p-2, pGEX-6p-3
Tn3.2	7–1216	B, C	116 221	pGEM-1 pGEM-2, pGEM-4, pGEM-Luc, pUC12, pUC13, pUC18, pUC19, pUC21, pUC57, pUC118, pUC119, pUCX
Tn3.3	77–1216	B	80 658	pET20b(+), pET21a-d(+), pET22(+), pET23a-d(+), pET25b(+), pET31b(+), pET32a-c(+), pET32 Ek/LIC, pET32 Xa/LIC,
Tn3.4	76–1216	B, C	77 342	pGEM-5, pGEM-5Zf(+), pGEM-T, pGEM-T easy vector, pGEMT-3P2A, pGEMT-PTE2A
Tn3.5	6–1216	B, C	27 508	pQE9, pQE16, pQE30, pQE31, pQE32, pQE40, pQE60, pQE70, pQE80-L, pQE81-L. pQE82-L
Tn3.6	7–1216	B, C, D	8930	pGEM-3Z, pGEM-4Z
Tn3.7	111–1171	B, C, O, P	6688	pETduet-1, pET43 Ek/LIC, pET43.1a(+), pET44a-c(+), pET45b(+), pET46 Ek/LIC, pET51b(+), pET51 Ek/LIC, pET52(+), pET52 Ek_LIC
Tn3.8	7–1216	C to M	5750	pGEM-3Zf(+), pGEM-3Zf(−), pGEM-11zf(+), pGEM-11Zf(−), pGEMEX-1, pGEMEX-2
Tn3.9	77–1216	C to M	3920	pGEM-7Zf(+), pGEM-7Zf(−)
Tn3.10	116–1174	C	2930	pBAD24
Tn3.11	116–1174		2758	pBAD18, pBAD30, pBAD-bHS, pBAD-EGFP
Tn3.12	116–1216	C	1601	pBAD7HisB-iRFP670, pBAD/HisD-TagRFP675, pBAD/Myc-HisA, pBAD/Myc-HisB, pBAD/Myc-HisC, pBAD/gii A, pBAD/gii-B, pBAD/gii-C, pBAD/gii/Calmod, pBAD/HisB
Tn3.13	77–1216	B, C	270	pGEM-5Zf(−), pGEM-9Zf(−)
Tn3.14	87–1216	B	167	pET303-CT-His-Rac Kinase, pET303-CT-His
Tn3.15	13–1216	B, C, N	104	pUCsg-RNA, pUCC001
Tn3.16	113–1216	B	64	pBAD-DEST49, pBAD/Myc-His/LacZ, pBAD/D-TOPO
Tn3.17	113–1161	O	23	pBAD18s

a A: 5′TTCTTGAAGACGAAAGGGCCTCGTGATACGCCTATTTTTATAGGTTAATGTCATGATAATAATGGTTTCTTAGACGTCAGGTGGCACTTTTCGGGGAAATGTGCGCGGAACCCCTATTTGTTTATTTTTCTAAATACATTCAAATATGTATCCGCTCATGAGACAATAACCCTGATAAATGCTTCAATAATATTGAAAAAGGAAGAGTATGAGTATTCAACATTTCCGTGTCGCCCTTATTCCCTTTTTTGCGGCATTTTGCCTTCCTGTTTTTGCTCACCCAGAAACGCTGGTGAAAGTAAAAGATGCTGAAGATCAGTTGGGTGCACGAGTGGGTTACATCGAACTGGATCTCAACAGCGGTAAGATCCTTGAGAGTTTTCGCCCCGAAGAACGTTTTCCAATGATGAGCACTTTTAAAGTTCTGCTATGTGGCGCGGTATTATCCCGTGTTGACGCCGGGCAAGAGCAACTCGGTCGCCGCATACACTATTCTCAGAATGACTTGGTTGAGTACTCACCAGTCACAGAAAAGCATCTTACGGATGGCATGACAGTAAGAGAATTATGCAGTGCTGCCATAACCATGAGTGATAACACTGCGGCCAACTTACTTCTGACAACGATCGGAGGACCGAAGGAGCTAACCGCTTTTTTGCACAACATGGGGGATCATGTAACTCGCCTTGATCGTTGGGAACCGGAGCTGAATGAAGCCATACCAAACGACGAGCGTGACACCACGATGCCTGCAGCAATGGCAACAACGTTGCGCAAACTATTAACTGGCGAACTACTTACTCTAGCTTCCCGGCAACAATTAATAGACTGGATGGAGGCGGATAAAGTTGCAGGACCACTTCTGCGCTCGGCCCTTCCGGCTGGCTGGTTTATTGCTGATAAATCTGGAGCCGGTGAGCGTGGGTCTCGCGGTATCATTGCAGCACTGGGGCCAGATGGTAAGCCCTCCCGTATCGTAGTTATCTACACGACGGGGAGTCAGGCAACTATGGATGAACGAAATAGACAGATCGCTGAGATAGGTGCCTCACTGATTAAGCATTGGTAACTGTCAGACCAAGTTTACTCATATATACTTTAGATTGATTTAAAACTTCATTTTTAATTTAAAAGGATCTAGGTGAAGATCCTTTTTGATAATCTCATGACCAAAATCCCTTAACGTGAGTTTTCGTTCCACTGAGCGTCAGACCCC-3′.

B: G244 to A mutation in *bla* (V82 to I).

C: C545 to T mutation in *bla* (A182 to V); deletion of the *Pst*I site.

D: G553 to C mutation in *bla* (A185 to P).

E: G226 to C, G227 to A, C228 to T mutations in *bla* (G76 to H).

F: G229 to A, G231 to A mutations in *bla* (A77 to T).

G: G232 to C mutation in *bla* (V78 to L).

H: G244 to A mutation in *bla* (G82 to H).

I: C275 to G mutation in *bla*.

J: A276 to G mutation in *bla*.

K: T277 to C mutation in *bla* (I93 to A).

L: A278 to G mutation in *bla* (I93 to A).

M: C281 to G mutation in *bla* (H94 to R).

N: G717 to T mutation in *bla*.

O: Nucleotide mutation in 5′UTR (−20 A to C).

P: Nucleotide mutation in 5′UTR (−93 A to C).

Q: Length relative to Tn3.1.

R: Nucleotide sequence of the Tn3.1 fragment is indicated by a B–P. Nucleotide changes from the Tn3.1 fragment are indicated by another letter. Numbering as depicted in Figure 1A, where the A of the AUG start codon for *bla* is denoted as +1.

S: Obtained from Google Scholar.

A widely acknowledged problem with Tn3.1-based fragments is that they produce excessively high levels of β-lactamase.^10^ As a result, cells are resistant to concentrations of β-lactam antibiotics that exceed the concentration required for selection. For example, most laboratory strains of *E. coli* are susceptible to <3 μg/mL of ampicillin. But when they harbor a medium-copy-number plasmid containing the Tn3.1 fragment, they are resistant to >5000 μg/mL.^14^ High-level production of β-lactamase contributes to the complete degradation of β-lactam antibiotics in culture media within 3 h, leading to loss of selective pressure.^9−13^ Cells that have lost the plasmid, as well as contaminating strains, can dominate the culture in the absence of a selection pressure.^9,11,13,15,16^ These problems could all be mitigated by reducing the production levels of β-lactamase from the Tn3-based cassettes.

High production levels of the TEM-1 β-lactamase from Tn3.1-based fragments are largely dependent on the efficiency of *bla* transcription and translation. Transcriptional initiation is mediated by the RNA polymerase of the host cell at the constitutive P3 promoter.^6^ Translational initiation is mediated by the 30S subunit of the ribosome at the translation initiation region (TIR),^17^ a stretch of approximately 30 nucleotides composed of the Shine–Dalgarno sequence, a linker region of five nucleotides, and the first five or six codons of the coding sequence.^18^ Production levels of the TEM-1 β-lactamase from Tn3.1-based fragments are also influenced by the copy number of the expression plasmid containing it.^19,20^ Herein, we have attempted to circumvent the problems associated with high-level production of β-lactamase by engineering a constitutively low-expressing version of the Tn3.1 fragment. We describe and characterize Tn3.1^MIN^, which differs from Tn3.1 by only four nucleotides in the TIR and which addresses all of the previously described problems with the Tn3.1-based fragments.

## Results

### The Tn3.1 Fragment Confers Resistance to High Concentrations of β-Lactam Antibiotics

**Figure 2:**
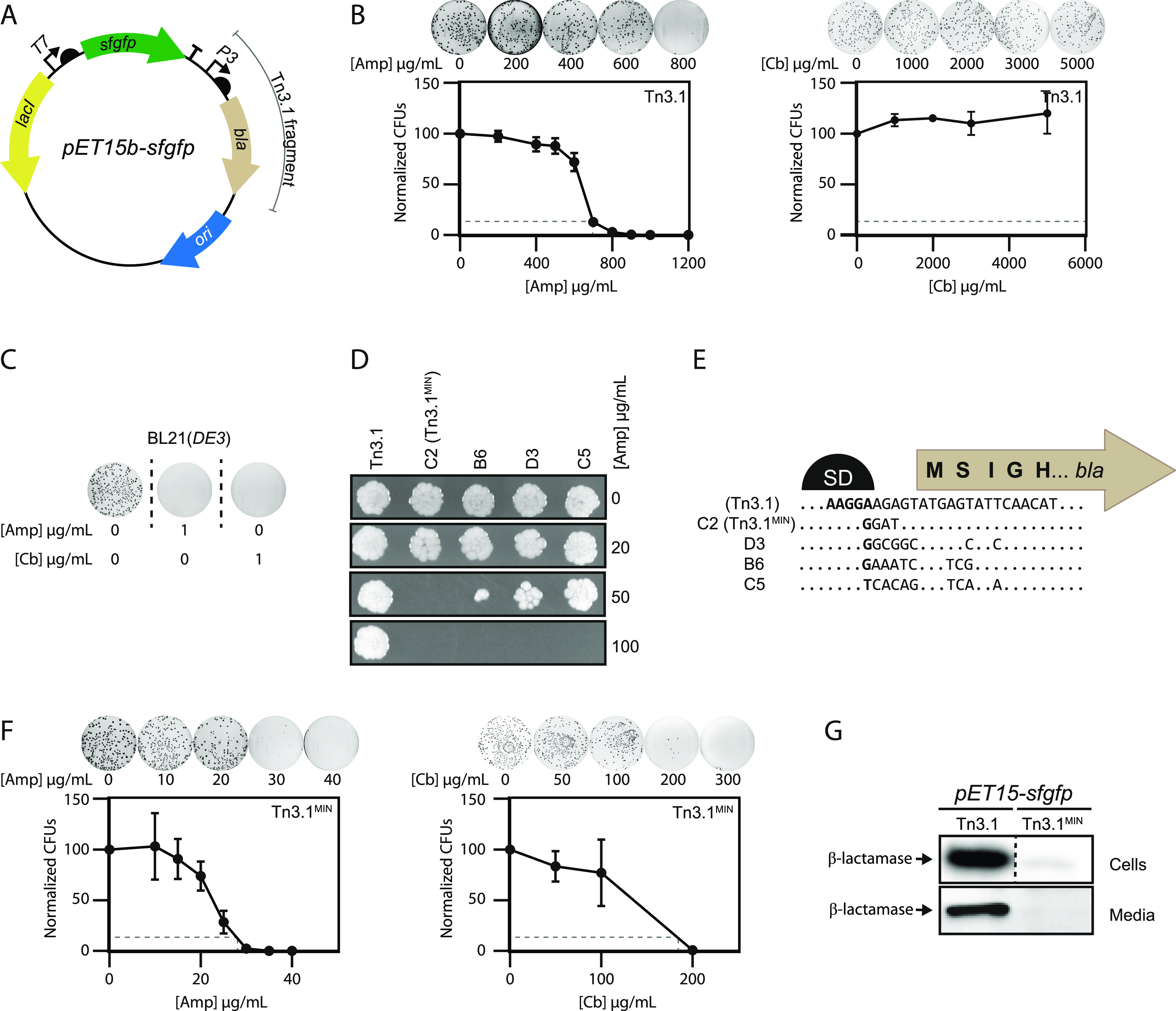
Tn3.1^MIN^ reduces levels of the TEM-1 β-lactamase. (A) Illustration of the *pET15b-sfgfp* expression plasmid, which contains the Tn3.1 fragment. (B) BL21(*DE3*) harboring the *pET15b-sfgfp* (Tn3.1) expression plasmid was plated on LB agar containing different concentrations of ampicillin or carbenicillin. Colony numbers were normalized by the number of colonies that grew in the absence of antibiotics. The minimum inhibitory concentration (MIC_90_) required to kill 90% of cells was extrapolated from the curve (dotted line) and deemed to be approximately 700 μg/mL for ampicillin and >5000 μg/mL for carbenicillin. (C) BL21(*DE3*) cells (without an expression plasmid) were plated on LB agar containing no antibiotic or 1 μg/mL ampicillin or carbenicillin. As growth was not observed on 1 μg/mL ampicillin or carbenicillin, the MIC_90_ was deemed to be <1 μg/mL. (D) BL21(*DE3*) harboring the *pET15b-sfgfp* expression plasmids plated on LB agar containing different concentrations of ampicillin. The *pET15b-sfgfp* expression plasmids, denoted C2, B6, D3, and C5, were selected from a directed evolution process and contained Tn3.1 fragments with a different translation initiation region (TIR) for *bla*. C2 was chosen for further characterization and was named Tn3.1^MIN^. (E) Nucleotide sequence alignment of the TIR for *bla* in Tn3.1, Tn3.1^MIN^ (C2), B6, D3, and C5. The TIR is defined as the nucleotide sequence from the Shine–Dalgarno (SD) region through to the fifth codon.^18^ (F) As in panel (B) except that BL21(*DE3*) harbored the *pET15b-sfgfp* (Tn3.1^MIN^) plasmid. The MIC_90_ was deemed to be <30 μg/mL for ampicillin and <200 μg/mL for carbenicillin. (G) Levels of TEM-1 β-lactamase in BL21(*DE3*) harboring the *pET15b-sfgfp* expression plasmid (Tn3.1 or Tn3.1^MIN^), or the culture media, were probed by Western blotting with antisera to the TEM-1 β-lactamase.

Initially, the *pET15b* expression plasmid was used, which contains the Tn3.1 fragment and the coding sequence for a polyhistidine-tagged super-folder green fluorescent protein (*pET15b-sfgfp*) (Figure 2A). *pET15b-sfgfp* was transformed into the BL21(*DE3*) strain, grown in liquid culture to mid-exponential phase and plated on lysogeny broth (LB) agar containing different concentrations of ampicillin or carbenicillin. Colony counting indicated that the minimum inhibitory concentration required to kill 90% of cells (MIC_90_) was >700 μg/mL of ampicillin (Figure 2B, left panel) and >5000 μg/mL of carbenicillin (Figure 2B, right panel). These MIC_90_s are considerably higher than that needed for selection, as the MIC_90_ for the BL21(*DE3*) strain is <1 μg/mL for both ampicillin and carbenicillin (Figure 2C). A similar observation was made when using the BL21(*DE3*) *pLysS* strain (Figure S1, Supporting Information (SI)). These data support previous observations^14^ indicating that the Tn3.1 fragment confers resistance to excessively high concentrations of β-lactam antibiotics.

### Reduced Production of the TEM-1 β-Lactamase from the Tn3.1 Fragment

We reasoned that the excessively high levels of resistance to β-lactam antibiotics are caused by high production titers of the TEM-1 β-lactamase from the Tn3.1 fragment. We utilized a directed evolution approach to identify a translation initiation region (TIR) that supported lower production yields, while retaining the constitutive *P3* promoter. This approach was used as it gives a wide range of expression levels from a relatively small sequence library.^21^

Four new TIRs for the TEM-1 β-lactamase were selected from the library by plating on different concentrations of ampicillin. All reduced the level of resistance to below 100 μg/mL ampicillin (Figure 2D). The one conferring the lowest level of resistance (denoted C2) was chosen for further characterization. This TIR had four nucleotide changes upstream of the *AUG* start codon, which most likely changed the Shine–Dalgarno sequence (Figure 2E). Colony counting indicated that the MIC_90_s of BL21(*DE3*) harboring *pET15b-sfgfp* were reduced to <30 μg/mL of ampicillin (Figure 2F, left panel) and <200 μg/mL of carbenicillin (Figure 2F, right panel). Similar observations were made when using the BL21(*DE3*) pLysS strain (Figure S1, SI). Western blotting indicated that β-lactamase levels in both the cells and the media were reduced considerably (Figure 2G). Taken together, these data indicate that β-lactamase production levels from the Tn3.1 fragment can be reduced by the selection of a new TIR. We refer to the new version of the genetic cassette as Tn3.1^MIN^ (MINimal production). Based on the MIC_90_s that we observed, we suggest that Tn3.1^MIN^, when integrated into medium-copy-number plasmids such as *pET15b*, are selected for at a concentration of 20 μg/mL of ampicillin and carbenicillin. These concentrations are 5-fold lower than that normally used for plasmid maintenance and >20-fold higher than that needed for selection against the BL21(*DE3*) strain lacking the plasmid.

### Tn3.1^MIN^ Increases the Half-Life of Antibiotics

**Figure 3:**
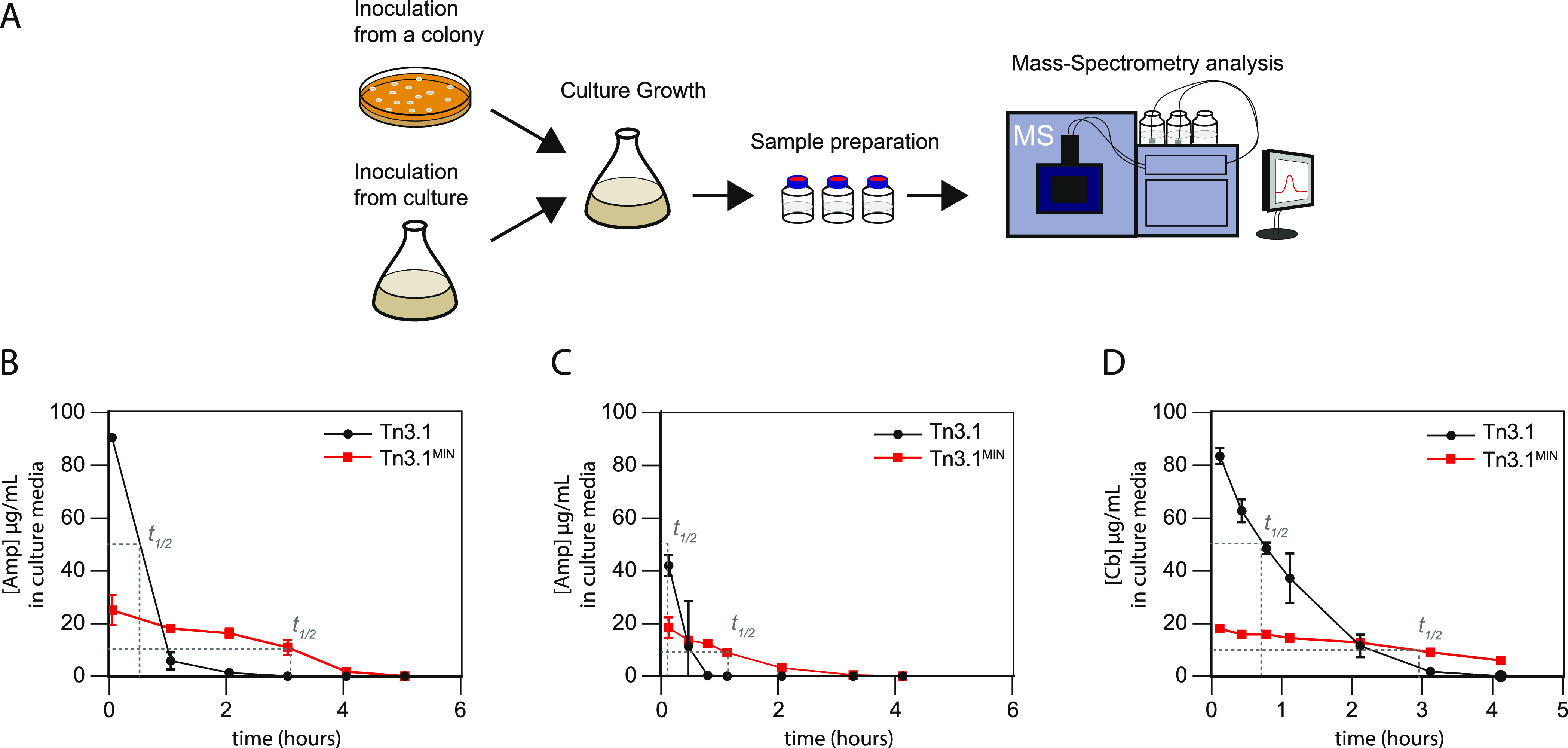
Tn3.1^MIN^ increases the half-life of ampicillin and carbenicillin in the culture media. (A) Schematic of the experimental workflow used to assess the concentration of ampicillin and carbenicillin in the culture media. Either a single colony or an overnight culture was used to inoculate fresh LB media containing either 100 μg/mL (Tn3.1) or 20 μg/mL (Tn3.1^MIN^) ampicillin or carbenicillin. Aliquots were analyzed using a semiquantitative mass spectrometry approach. (B) Concentration of ampicillin in the culture media when a single colony of BL21(*DE3*) harboring *pET15b-sfgfp* was inoculated. Concentrations are plotted against culture time. (C) As for panel (B) except that an overnight culture was back-diluted 1:100. (D) As for panel (C) except that carbenicillin was used.

A widely acknowledged problem with the Tn3.1-based cassettes is that the exceedingly high titers of β-lactamase cause rapid degradation of β-lactam antibiotics in culture media.^9−11,13^ This in turn leads to loss of selective pressure. As Tn3.1^MIN^ reduced the production titers of β-lactamase, we were curious to know if the half-life of antibiotics increased. A single colony of BL21(*DE3*) harboring *pET15b-sfgfp* was inoculated into LB media containing ampicillin (20 μg/mL for Tn3.1^MIN^ and 100 μg/mL for Tn3.1) and a semiquantitative mass spectrometry (MS) assay was used to monitor the concentration of ampicillin in the culture media during cultivation (Figure 3A). Rapid degradation of ampicillin was observed when the Tn3.1 cassette was integrated in *pET15b-sfgfp*. The *t*_1/2_ was calculated to be approximately 30 min, and the culture media was deemed to be above 1 μg/mL (the concentration required for selection) for 130 min (Figure 3B). When Tn3.1^MIN^ was integrated in *pET15b-sfgfp*, the *t*_1/2_ was calculated to be approximately 3 h, and the culture media was deemed to be above 1 μg/mL for approximately 250 min (Figure 3B).

When overnight cultures of BL21(*DE3*) harboring *pET15b-sfgfp* were back-diluted 1:100 into LB media containing ampicillin or carbenicillin, as is typical for an experiment, we observed even shorter *t*_1/2_’s. When the Tn3.1 cassette was integrated in *pET15b-sfgfp*, the *t*_1/2_ of ampicillin was calculated to be 6 min, and the culture media was deemed to be above 1 μg/mL for approximately 70 min (Figure 3C). When Tn3.1^MIN^ was integrated in *pET15b-sfgfp*, the *t*_1/2_ of ampicillin was calculated to be 63 min, and the culture media was deemed to be above 1 μg/mL for approximately 180 min (Figure 3C). When carbenicillin was used at the same concentrations, the *t*_1/2_ increased from 45 min (Tn3.1) to 175 min (Tn3.1^MIN^) (Figure 3D). These experiments indicate that the *t*_1/2_ of β-lactam antibiotics in the culture media was increased by 3–10-fold when using Tn3.1^MIN^, even though a 5-fold lower starting concentration was used.

### Tn3.1^MIN^ Helps Select for Cells That Harbor the Plasmid

**Figure 4:**
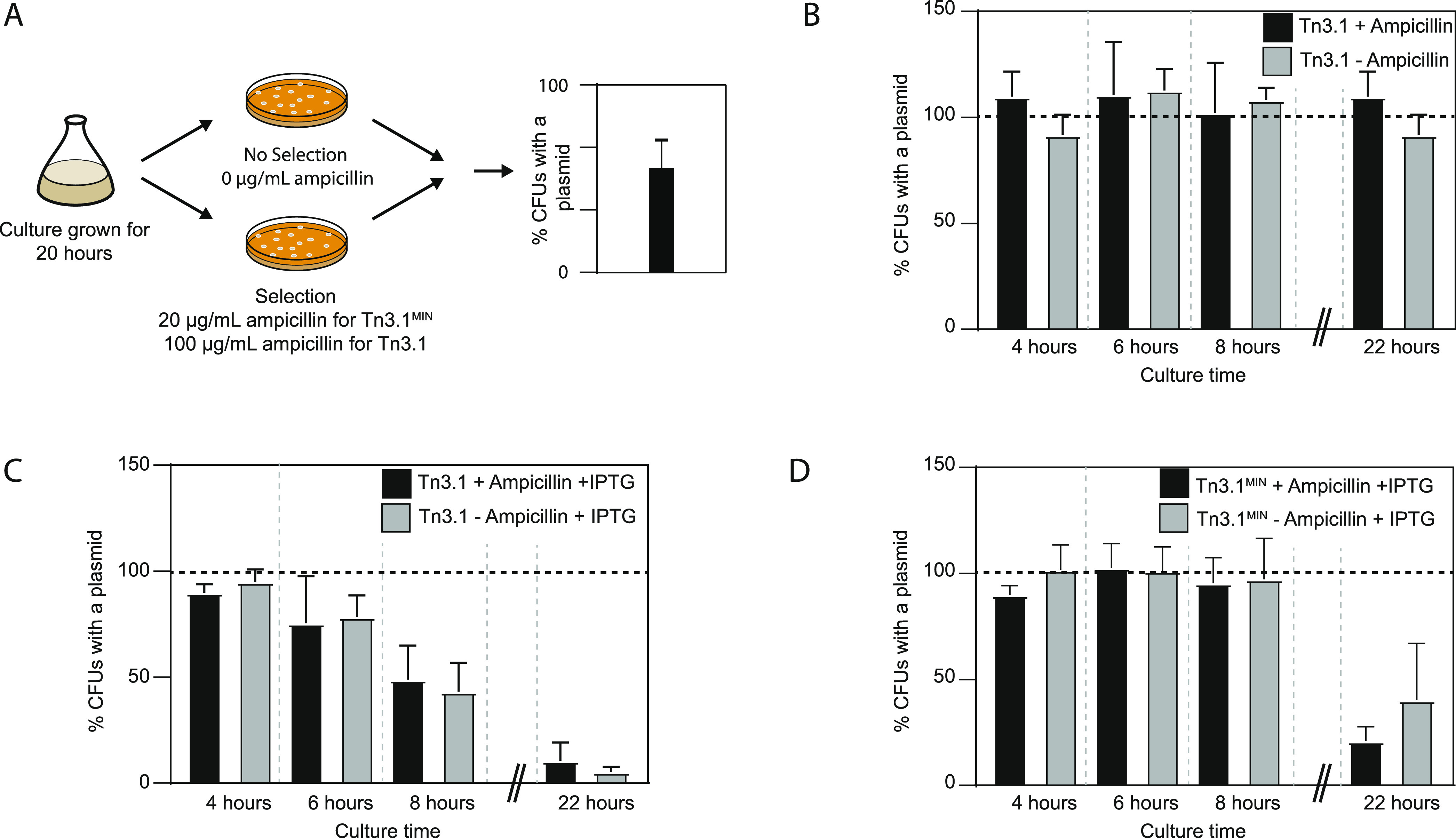
Tn3.1^MIN^ helps cells to maintain the plasmid. (A) Schematic representation of the experimental workflow for determining the percentage of cells with a plasmid. This was determined by calculating the relative ratio of colonies on LB agar plates, with or without ampicillin selection. (B) Percentage of BL21(*DE3*) harboring *pET15b-sfgfp* (Tn3.1). In cultures with and without ampicillin, most cells maintained a plasmid after 20 h of cultivation. (C) After induction of sfGFP with IPTG, the majority of BL21(*DE3*) did not have the *pET15b-sfgfp* (or Tn3.1) plasmid. (D) As for panel (C) except that the *pET15b-sfgfp* (or Tn3.1^MIN^) plasmid was used. Here a larger proportion of cells in the culture harbored the plasmid. Data presented as mean ± standard deviation (s.d.) (*n* ≥ 3).

In the absence of antibiotic selection, cells that have lost their plasmids during division can eventually dominate the culture.^22,23^ To investigate the rate of plasmid loss without antibiotic selection, cultures were plated on LB agar with or without ampicillin and CFU’s were compared (Figure 4A). When BL21(*DE3*) harboring the *pET15b-sfgfp* (Tn3.1) plasmid was cultured in the presence of 100 μg/mL ampicillin, we observed that almost all of the cells in the culture maintained the plasmid over a 22-h period (Figure 4B). The same observation was made when ampicillin was omitted from the culture (Figure 4B). When the same experiment was repeated, this time with the addition of 0.5 mM isopropyl β-d-1-thiogalactopyranoside (IPTG) to induce production of sfGFP (after 2 h of culturing), we observed that cells without the plasmid started to accumulate in the culture. And after 6 h of induction (8 h of culturing) approximately 50% of the cells in the culture did not have the plasmid. After 22 h of culturing, almost none of the cells in the culture had maintained the plasmid (Figure 4C). The same observation was made when ampicillin was omitted from the culture (Figure 4C). These data indicate that recombinant protein production results in an accumulation in plasmid-free cells within the time frame of a standard protein expression experiment.

To determine if Tn3.1^MIN^ could mitigate this problem, the same experiment was replicated with BL21(*DE3*) harboring the *pET15b-sfgfp* (Tn3.1^MIN^). It was observed that almost all of the cells in the culture maintained the plasmid after 6 h of induction (8 h of culturing). After 22 h of culturing, the majority of the cells did not contain a plasmid (Figure 4D). The same observations were made when ampicillin was omitted from the culture. These data indicate that the increased *t*_1/2_ of ampicillin was not the factor contributing to better plasmid maintenance in the culture. Nevertheless, Tn3.1^MIN^ lowers the rate at which cells without a plasmid dominate the culture.

To determine whether the type of recombinant protein being produced had an impact on plasmid loss, a human protein that was soluble in the cytoplasm of *E. coli* (Mth1) and another that is known to form inclusion bodies (Neil3)^24^ were expressed. When the soluble Mth1 was produced, more cells with a plasmid were observed with Tn3.1^MIN^ after 20 h of induction (8% for Tn3.1 *vs* 18% for Tn3.1^MIN^). When the inclusion body-prone Neil3 was achieved, cells without a plasmid completely dominated both cultures and differences between Tn3.1 and Tn3.1^MIN^ could not be resolved (Figure S2, SI). Although plasmid loss was mitigated by Tn3.1^MIN^ when sfGFP and Mth1 were produced, we did not observe any differences in production levels for these recombinant proteins (Figure S3, SI).

### Tn3.1^MIN^ Prevents Contamination

**Figure 5:**
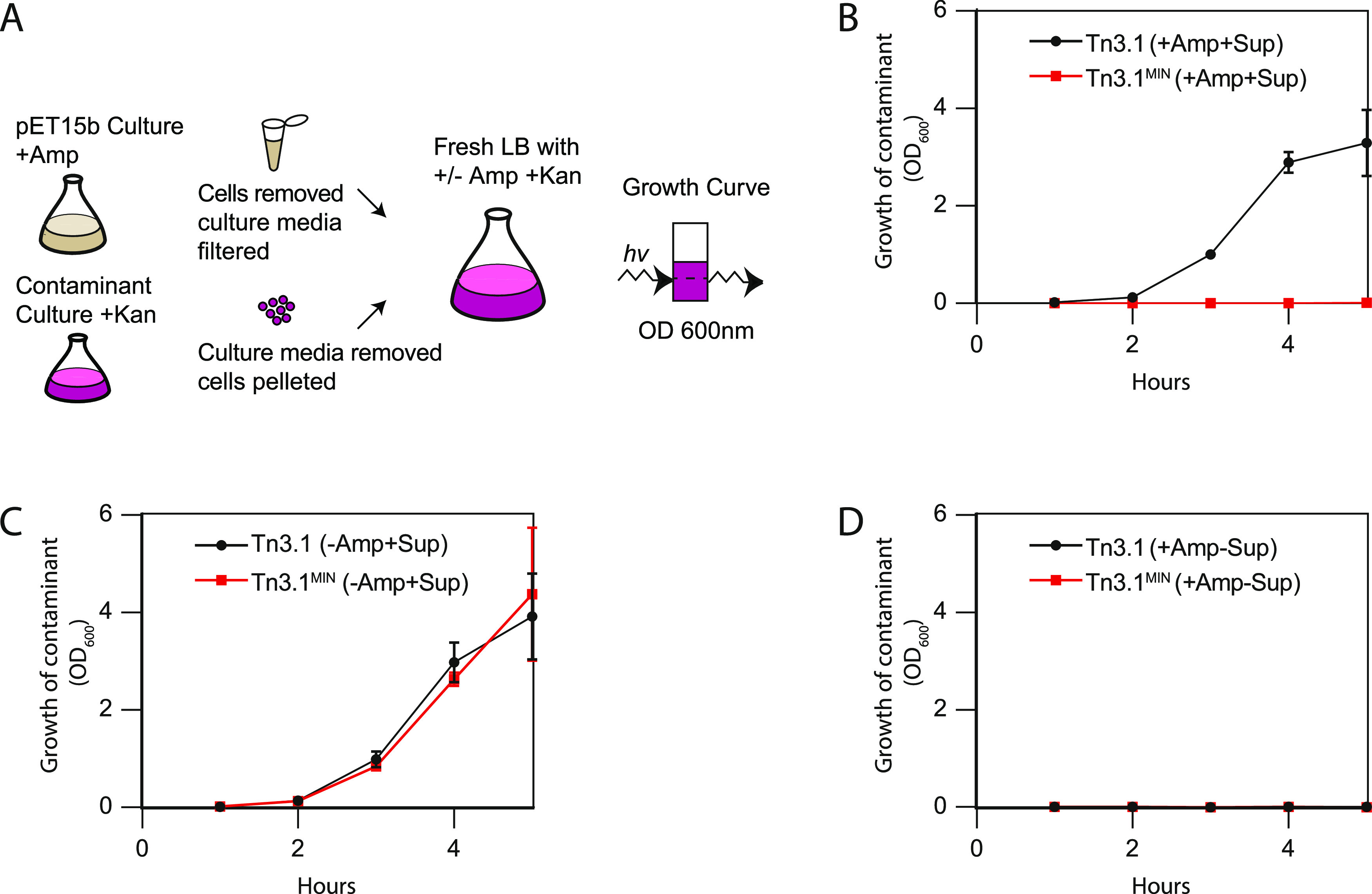
Tn3.1^MIN^ prevents contamination. (A) Schematic representation of the experimental workflow for determining whether contaminants can survive after back-dilution. Supernatants from overnight cultures of BL21(*DE3*) harboring *pET15b-sfgfp* were back-diluted in the presence of a contaminant; BL21(*DE3*) harboring *pET28a-mcherry*. Growth of the contaminant was monitored by cell density. (B) Growth of the contaminant was monitored in LB media containing ampicillin (100 μg/mL for Tn3.1 or 20 μg/mL for Tn3.1^MIN^). The contaminant could grow when Tn3.1 was used but not Tn3.1^MIN^. (C) As for panel (B) except that ampicillin was omitted in the back-dilution. This control indicates that the contaminant can always grow in the absence of ampicillin, when the supernatants are present. (D) As for panel (B) except that the supernatants were omitted in the back-dilution. This control indicates that the contaminants cannot grow in the presence of ampicillin.

Back-diluted overnight cultures contain high titers of β-lactamase in the media that can degrade ampicillin directly as shown in Figure 3C. As a result, it is possible that cells without a plasmid, as well as contaminants, have a chance to grow and become dominant in the cell population.^9,11,15,16^ This possibility was explored by collecting the culture media from overnight cultures of BL21(*DE3*) harboring *pET15b-sfgfp* (Tn3.1 and Tn3.1^MIN^), and then back-diluting it 1:100 (without cells) into fresh LB media containing ampicillin (20 μg/mL for Tn3.1^MIN^ and 100 μg/mL for Tn3.1). An ampicillin-sensitive “contaminant” was then spiked into the fresh LB media and its growth was monitored (Figure 5A). Although the contaminant should not be able to grow in the presence of ampicillin, growth was observed when media from a *pET15b-sfgfp* (Tn3.1) culture was back-diluted (Figure 5B). Growth of the contaminant was not observed when media from a *pET15b-sfgfp* (Tn3.1^MIN^) culture was back-diluted, indicating that concentrations of ampicillin remained above 1 μg/mL (needed for selection) long enough to eliminate the contaminant cells (Figure 5B). To ensure that the growth inhibition was not caused by metabolites in the spent media, ampicillin was omitted and subsequently growth was observed at an equivalent rate to when no supernatant was added (Figure 5C). Growth was not observed in the absence of supernatant when ampicillin was added at either 20 or 100 μg/mL (Figure 5D). These data indicate that the high levels of TEM-1 β-lactamase in the overnight growth media when using Tn3.1 result in degradation of the ampicillin so fast that contaminating cultures are not being selected against. This problem is mitigated by Tn3.1^MIN^.

### Next-Generation β-Lactamase Cassette for the pSEVA Series

**Figure 6:**
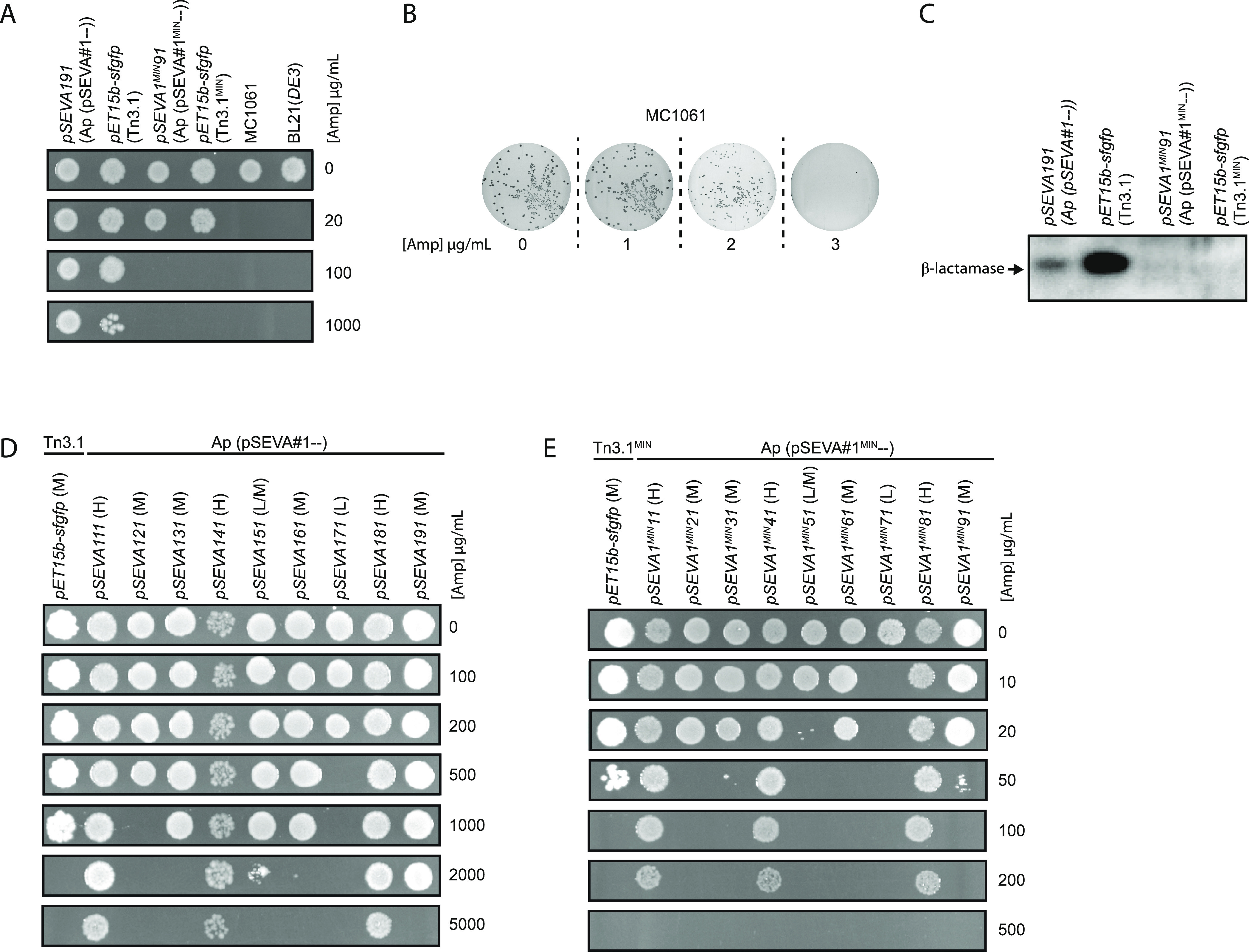
Characterization of Ap (pSEVA#1^MIN^--). (A) MC1061 cells harboring the *pSEVA1*91 or *pSEVA1*^*MIN*^*91* plasmid were spotted on LB agar plates with different concentrations of ampicillin. For comparison, BL21(*DE3*) harboring the *pET15b-sfgfp* (Tn3.1 and Tn3.1^MIN^) expression plasmid was also spotted. When Ap (pSEVA#1--) and Tn3.1 were used, cells could survive on 1000 μg/mL of ampicillin. When Ap (pSEVA#1^MIN^--) and Tn3.1^MIN^ were used, cells could only survive on 20 μg/mL of ampicillin. (B) MC1061 cells (without an expression plasmid) were plated on LB agar containing 0, 1, 2, and 3 μg/mL ampicillin. As growth was not observed on 3 μg/mL ampicillin, the MIC_90_ was deemed to be <3 μg/mL. (C) Levels of TEM-1 β-lactamase were probed by Western blotting with antisera to the TEM-1 β-lactamase. β-Lactamase could only be observed when Ap (pSEVA#1--) and Tn3.1 were used. (D) Collection of *pSEVA* plasmids with Ap (pSEVA#1--) and different origins of replication were transformed into MC1061 cells and spotted on LB agar plates with different concentrations of ampicillin. For comparison, BL21(*DE3*) harboring the *pET15b-sfgfp* (Tn3.1) expression plasmid was also spotted. “L” denotes a low copy, “M” a medium copy, and “H” a high copy plasmid. (E) As in panel (D) except that all pSEVA vectors contained Ap (pSEVA#1^MIN^--) and pET15b-sfgfp contained Tn3.1^MIN^.

The Standard European Vector Architecture (SEVA) contains a genetic cassette for β-lactamase, the Ap (pSEVA#1--) module, which differs from the Tn3.1 fragments in most other common plasmids (Figure 1). To determine if the Ap (pSEVA#1--) module also conferred high-level resistance to ampicillin, it was cloned with a *pBR322* origin of replication to generate a *pSEVA1*91 plasmid. *pSEVA191* was then transformed into the MC1061 strain and spotted onto LB agar plates containing different concentrations of ampicillin (Figure 6A). Growth was observed on 1000 μg/mL ampicillin, which is more than 300 times higher than that needed to select against the MC1061 strain (Figure 6B). For comparison, cells containing the *pET15b-sfgfp* plasmid, which contained Tn3.1, also grew on a concentration of 1000 μg/mL ampicillin (albeit not as well). Western blotting of whole cells indicated that expression of β-lactamase from the Ap (pSEVA#1--) module was not as high as it was from the Tn3.1 fragment in the *pET15b-sfgfp* plasmid, but it was still easily detectable (Figure 6C). We therefore presume that the problems associated with high-level production of β-lactamase (as outlined above) are applicable to the Ap (pSEVA#1--) module.

To circumvent these problems the Ap (pSEVA#1--) module was re-engineered so that it resembled the Tn3.1^MIN^ fragment. Here the region from the 5′-end of the P3 promoter through to the stop codon of the codon-optimized *bla* was removed from the Ap (pSEVA#1--) module and replaced by the corresponding region from the Tn3.1^MIN^ fragment. The transcriptional terminators and flanking restriction sites from Ap (pSEVA#1--) were retained. The new module is referred to as Ap (pSEVA#1^MIN^--). When cloned with a *pBR322* origin of replication to generate the *pSEVA1*^*MIN*^*91* plasmid, we observed growth on 20 μg/mL ampicillin but not 100 μg/mL (Figure 6A). This level of resistance is considerably reduced compared to the *pSEVA191* plasmid. Western blotting indicated that the Ap (pSEVA#1^MIN^--) module reduced the expression of β-lactamase to an undetectable level (Figure 6C).

We exploited the pSEVA collection to determine how the Ap (pSEVA#1^MIN^--) worked with other origins of replication. A collection of *pSEVA* plasmids was constructed with nine different origins of replication (Table S1, SI). This included low (*pSEVA1*71), medium (*pSEVA121, pSEVA131, pSEVA1*51*, pSEVA161, pSEVA191*) and high copy (*pSEVA111, pSEVA141, pSEVA181*) variants. Initially, the Ap (pSEVA#1--) module was integrated and the cells were spotted on different concentrations of ampicillin. As noted previously,^19,20^ the level of resistance to ampicillin roughly correlated with copy number (Figure 6D), and aside from the low-copy-number *pSEVA171*, the level of resistance to ampicillin was unnecessarily high. When the Ap (pSEVA#1^MIN^--) module was integrated in the same plasmids, the level of resistance to ampicillin decreased by >25-fold in all cases (Figure 6E). For the low-copy-number *pSEVA1*^*MIN*^*71* and the medium-copy-number *pSEVA1*^*MIN*^*51*, the level of resistance to ampicillin was <20 μg/mL, which is below the level we recommend. All other plasmids survived on more than 20 μg/mL of ampicillin. Moreover, the level of resistance to ampicillin conferred by the Ap (pSEVA#1^MIN^--) module roughly correlated with the copy number of the plasmids. Taken together, these data show that the Ap (pSEVA#1^MIN^--) module reduces β-lactamase expression levels, presumably mitigating the problems caused by excessive production. It can be used with medium and high copy origins of replication.

## Discussion

Genetic cassettes encoding the TEM-1 β-lactamase (the Tn3.1-based fragments) have been used as selection markers in bacterial expression plasmids for more than 50 years.^5^ During this time, these fragments have been propagated, either unchanged (Tn3.1) or with minor changes (Tn3.2–Tn3.17), into more than 120 commercially available expression plasmids (see Table 1). And these expression plasmids have been used in >581 000 published studies. A widely acknowledged flaw with the cassettes is that they produce excessive amounts of β-lactamase, which rapidly degrade β-lactam antibiotics in the culture media, leading to loss of selective pressure.^9−13^ And in the absence of selection pressure, cells that have lost the plasmid can dominate the culture.^22,23^ In this study, we describe and characterize a next-generation version of the genetic cassette, which we refer to as Tn3.1^MIN^ (MINimal expression). Tn3.1^MIN^ contains only four nucleotide changes in the TIR, which reduces the amount of β-lactamase in both the cell and in the culture media. As a consequence, the *t*_1/2_ of β-lactam antibiotics in the culture media is increased and selection pressure is maintained for a longer period.

It is widely assumed that antibiotic selection pressure is essential for maintaining an expression plasmid. Our study challenges this dogma, as we observed that most cells harbored a plasmid even after 22 h of culture in the absence of ampicillin. However, when recombinant protein production was induced, we observed that most cells in the culture no longer harbored a plasmid after 6 h (8 h of culture). We reason that recombinant protein production was slowing the growth of cells harboring a plasmid and giving those that had lost the plasmid a significant growth advantage. This growth advantage was reduced when Tn3.1^MIN^ was used and most cells harbored a plasmid after 6 h of induction (8 h of culture). Intriguingly similar results were observed in the absence of ampicillin; Thus, we reason that reduced expression of β-lactamase from the Tn3.1^MIN^ fragment contributed to more cells harboring a plasmid by reducing the metabolic load. The increased half-life of ampicillin appeared to have little bearing.

Tn3.1^MIN^ can be easily incorporated into expression plasmids that currently contain a Tn3.1 fragment by incorporating just four nucleotide changes in the translation initiation region. For those expression plasmids that currently contain a Tn3.2–Tn3.17 fragment, we reason that the same four nucleotide changes in the TIR would work similarly, but this has not been tested in this study. A more reliable approach would be to incorporate the entire 1216-nucleotide-long Tn3.1^MIN^ fragment, as its performance has been characterized here. For the Standard European Vector Architecture,^7,8^ we designed a novel fragment that was based on Tn3.1^MIN^, which we have called Ap (pSEVA#1^MIN^--). Both Tn3.1^MIN^ and Ap (pSEVA#1^MIN^--) confer a similar level of resistance to ampicillin (and carbenicillin).

Why should one include the Tn3.1^MIN^ or Ap (pSEVA#1^MIN^--) fragment in an expression plasmid? Through the characterization presented in this paper three main advantages were identified: (1) The Tn3.1^MIN^ fragment reduces antibiotic use by 5-fold. When the Tn3.1^MIN^ fragment was integrated into medium-copy-number expression plasmids the working concentration of β-lactam antibiotics was 5-fold lower than the concentration typically recommended (*i.e.*, 20 μg/mL instead of 100 μg/mL). This reduces antibiotic costs by 5-fold, which is particularly important if large culture volumes are used and/or if expression plasmids are used over long periods of time. Reducing antibiotic consumption is also an integral part of antibiotic stewardship and is being advocated by numerous healthcare and governmental bodies. (2) The Tn3.1^MIN^ fragment improves plasmid performance. Primarily it reduces the amount of β-lactamase in the cell and in culture media. As a consequence, the *t*_1/2_ of β-lactam antibiotics in the culture media is increased by 3- to 10-fold and selection pressure is maintained for a longer period. Metabolic load is presumably decreased, and the point at which plasmid-less cells overtake the culture is delayed. This is particularly important when recombinant proteins are being produced, as cells without a plasmid can quickly outgrow those with a plasmid. (3) The Tn3.1^MIN^ fragment improved selection against contaminants in the culture. When overnight cultures are back-diluted (1:100) they typically degrade β-lactam antibiotics so quickly that contaminants are able to grow.^9,11^ When a Tn3.1^MIN^ fragment was used, contaminants were unable to grow as the β-lactam antibiotics were not degraded sufficiently quickly.

It has been demonstrated that excessive production of antibiotic resistance proteins limits the cell’s capacity to produce a recombinant protein.^25^ Panayotatos and co-workers tested this hypothesis in *E. coli* by lowering the production of the neomycin phosphotransferase (which confers resistance to kanamycin) using a promoter mutagenesis approach.^14^ They observed that the production of one recombinant protein was increased by 2-fold, but that there was no improvement for the other. Although the Tn3.1^MIN^ fragment reduced the production of β-lactamase, and increased plasmid retention, we did not observe an increase in growth rate or in the production of the three recombinant proteins that were tested (*i.e.*, sfGFP, Mth1, Neil3). This discrepancy has not been addressed in the current study, but we speculate that it could be explained by suppressing mutations that downregulate the T7 polymerase and which may mask improvements in plasmid retention.^26^

Bacterial expression plasmids are widely used in both academia and industry.^27,28^ It is a poorly acknowledged fact that the genetic modules used to construct them were cloned and developed in the 1960s, 1970s, and 1980s, when methods in molecular biology were in their infancy and knowledge about protein biogenesis was less advanced than it is today. Recent work has identified design flaws in some of these genetic modules, which hinder their performance. “Next-generation” versions have been developed, for example, in promoters,^24,29^ standardized TIRs,^24,30^ transcriptional terminators,^31^ and origins of replication.^32^ These “next-generation” genetic modules outperform the original modules. The Tn3.1^MIN^ fragment developed here is an additional “next-generation” genetic module, which will contribute to making the expression plasmids and bacterial factories of the future more efficient.

## Methods

### Molecular Cloning

All polymerase chain reactions (PCR) were performed using the Q5 polymerase (New England Biolabs). All primers and DNA sequencing were carried out by Eurofins genomics (Germany). The *pET15b-sfgfp* expression plasmid was described in ref (24). The *pET15b-neil3*, *pET15b-mth1* expression plasmids were generated by PCR amplification of the coding sequences and ligation by *in vitro* assembly^33^ in the MC1061 strain (K-12 F^–^ λ^–^ Δ*(ara-leu)7697* [*araD139*]B/r Δ*(codB-lacI)3 galK16 galE15* e14^–^*mcrA0 relA1 rpsL150*(Str^R^) *spoT1 mcrB1 hsdR2*(*r*^–^*m*^+^)). *pSEVA111*, *pSEVA121*, *pSEVA131*, *pSEVA141, pSEVA151*, *pSEVA181*, *pSEVA191*, *pSEVA261*, and *pSEVA271* were generous gifts from Victor de Lorenzo and Esteban Martinez. *pSEVA161* and *pSEVA171* were generated by fragment shuffling from *pSEVA261* and *pSEVA271* into *pSEVA111* using the restriction enzymes *Box*I (*PshAI*) and *Smi*I (*Swa*I) (Thermo Fisher) as described in the SEVA system.^7^ The Ap (pSEVA#1--) fragment was generated by removing the region from the start of the *P3* promoter to the stop codon of *bla* and replacing it with the analogous region from Tn3.1^MIN^. This process was also carried out by PCR amplification of the coding sequences and ligation by *in vitro* assembly.^33^ A list of plasmids is available in Table S1 and primers in Table S2.

### Minimum Inhibitory Concentrations (MICs)

A single colony of MC1061, BL21(*DE3*) (B F^–^*ompT gal dcm lon hsdS*_*B*_(*r*_*B*_^–^*m*_*B*_^–^) λ(DE3 [*lacI lacUV5*-*T7p07 ind1 sam7 nin5*]) [*malB*^+^]_K-12_(λ^S^)) or BL21(*DE3*) *pLysS* (B F^–^*ompT gal dcm lon hsdS*_*B*_(*r*_*B*_^–^*m*_*B*_^–^) λ(DE3 [*lacI lacUV5*-*T7p07 ind1 sam7 nin5*]) [*malB*^+^]_K-12_(λ^S^) pLysS[*T7p20 ori*_p15A_](Cm^R^)) was inoculated into 5 mL of LB media with relevant antibiotics (Table S1) and incubated overnight with shaking at 37 °C. The cultures were then back-diluted 1:100 in fresh LB with relevant antibiotics in a 5 mL 24-well plate and grown to an OD_600_ between 0.3 and 0.7. The cultures were then serial diluted 1:10 000 (BL21(*DE3*) +/–*pLysS*) or 1:1 000 000 (MC1061) and 100 μL was plated onto LB agar plates with different concentrations of either ampicillin (Avantor) or carbenicillin (Formedium, U.K.). When BL21(*DE3*) *pLysS* cells were used, the plates also contained chloramphenicol (Alfa Aesar) at a concentration of 34 μg/mL. Images were taken using the upper white light in a GenoPlex (VWR International), and colonies were counted using OpenCFU.^34^ The MIC of antibiotic required to kill 90% of cells (MIC_90_) was determined from the colony numbers.

### Spot Assays

A single colony of MC1061 was inoculated into 5 mL of LB media with relevant antibiotics (Table S1) and incubated overnight with shaking at 37 °C. The cultures were then back-diluted 1:100 in fresh LB with relevant antibiotics in a 5 mL 24-well plate and grown to an OD_600_ between 0.3 and 0.7. The cultures were then serially diluted 1:100 and 1 μL of each culture was spotted onto LB agar plates with different concentrations of ampicillin. Survival was deemed to be the highest concentration of ampicillin on which growth was observed.

### Directed Evolution of the Translation Initiation Region (TIR)

The TIR for the gene encoding β-lactamase in *pET15b-sfgfp* was optimized using a directed evolution approach described previously.^35,36^ Briefly, forward and reverse degenerate primers were designed that allowed for all sequence possibilities in the six nucleotides preceding the *AUG* start codon, and restrained sequence possibilities (synonymous codon changes only) in the six nucleotides following the *AUG* start codon (Table S2, SI). The forward and reverse primers were overlapping by 18 nucleotides so that the subsequent PCR product could be re-ligated into a circular plasmid by *in vivo* assembly.^33^ Plasmid libraries with randomized TIRs were generated by PCR using the degenerate primers. The PCR cycle comprised 30 cycles of 95 °C for 30 s, 50 °C for 30 s, and 72 °C for 210 s. The resulting PCR product was treated with *Dpn*I then transformed into MC1061 cells and grown overnight at 37 °C. The plasmid library was purified using an Omega Bio-Tek mini-prep kit.

The purified plasmid library was transformed into BL21(*DE3*) cells and susceptibility to ampicillin was determined on LB agar plates. Forty-eight random colonies were inoculated into 500 μL of LB media in a 96-well 2.2 mL growth plate containing 20 μg/mL of ampicillin. Cultures were grown until an OD_600_ of approximately 0.3, and 2 μL of the culture was then spotted on LB agar plates containing 20, 50, 100, 200, 300, and 400 μg/mL of ampicillin. Four colonies that survived only on <100 μg/mL of ampicillin were selected. The plasmids were purified and the TIR for the gene encoding β-lactamase was sequenced. One plasmid was selected for further characterization and was therefore sequenced to 97.4%. Aside from mutations in the TIR, no mutations were identified in the plasmid backbone.

### Protein Expression

A single colony of BL21(*DE3*) harboring *pET15b-sfgfp, pET15b-mth1, or pET15b-neil3* (Tn3.1 and Tn3.1^MIN^) were grown in LB media supplemented with appropriate antibiotics for 16–20 h at 37 °C with shaking. Thereafter, the overnight cultures were back-diluted 1: 100 in 5 mL 24-well plates and grown at 37 °C with shaking to an OD_600_ of 0.3–0.7. The cultures were then induced with 0.5 mM IPTG. The cultures were then incubated for 20 h at 37 °C with shaking.

### Trichloroacetic Acid (TCA) Precipitation

TCA precipitations were carried out using a modified version of the method described in ref (37). In short, culture supernatants were mixed in a 1:4 ratio with 100% cold TCA and incubated on ice for 2 h, with occasional mixing. The precipitate was pelleted by centrifugation at 12 000*g* for 20 min, then washed in 200 μL of acetone—by resuspension, and then re-centrifugation at 17 000*g* for 20 min. The acetone was removed and the pellet was resuspended in sodium dodecyl sulfate-polyacrylamide gel electrophoresis (SDS-PAGE) loading buffer.

### SDS-PAGE and Western Blotting

SDS-PAGE was carried out on Tris-glycine 12% acrylamide and cast with a thickness of 1 mm. All were run using the Hoefer SE260 Mighty Small II Deluxe Mini Vertical Protein Electrophoresis Unit at 100 V for 3 h. For Western blotting, proteins were transferred onto a nitrocellulose membrane using a semidry Trans-Blot SD cell (Bio-Rad) for 1 h at 15 V. The nitrocellulose membranes were then incubated for either 1 h or overnight in 5% (w/v) nonfat milk (PanReac AppliChem) in Tris-buffered saline (TBS) (50 mM Tris, pH 7.4, 200 mM NaCl). His-tagged recombinant proteins were probed using the HisProbe-HRP conjugate (15165, Thermo Scientific) at a dilution of 1:10 000. β-Lactamase was probed using a mouse monoclonal primary antibody ((8A5.A10): sc-66062, Santa Cruz Biotechnology) at a 1:1000 dilution. For detection, a secondary anti-IgG Sheep Polyclonal Antibody HRP conjugate (NXA931, GE Healthcare) was used at a 1:3000 dilution. The SuperSignal West Pico PLUS Chemiluminescent Substrate (Thermo Scientific) was used as the substrate. Images were captured on an Azure c600 Imaging System (Azure Biosystems).

### Semiquantitative Analysis of Ampicillin and Carbenicillin in LB Media by Electrospray Ionization Tandem Mass Spectrometry (MS/MS)

#### Growth Conditions

A single colony of BL21(*DE3*) harboring *pET15b-sfgfp* (TN3.1 or TN3.1^MIN^) was grown in 5 mL LB media supplemented with ampicillin or carbenicillin (100 μg/mL for TN3.1 and 20 μg/mL for TN3.1^MIN^) for 16–20 h at 37 °C with agitation at 185 rpm. Thereafter, the overnight cultures were back-diluted 1:100 in 5 mL of LB media, in 24-well plates and grown at 37 °C with agitation at 185 rpm. A 250 μL sample of each culture was taken for analysis at 0, 20, 40, 60, 120, 180, 240, and 300 min (see Sample Work-Up).^38^ Alternatively, a single colony of BL21(*DE3*) harboring *pET15b-sfgfp* (TN3.1 or TN3.1^MIN^) was grown in 5 mL of LB media supplemented with ampicillin (100 μg/mL for TN3.1 and 20 μg/mL for TN3.1^MIN^) and samples were taken (without back-dilution) for analysis at 0, 60, 120, 180, 240, 300, and 360 min. Proteins were removed in the same manner as above.

#### Sample Work-Up

Cells were pelleted from a 250 μL sample of each culture by centrifugation (14 000*g*, 1 min). A 200 μL aliquot of the supernatant was mixed with 20 μL of an internal standard (IS) dissolved in water (1 μg/mL carbenicillin for ampicillin analysis, or 0.1 μg/mL ampicillin for carbenicillin analysis). A 380 μL aliquot of acetonitrile was added immediately, the sample was shaken, and centrifuged at 17 000*g* for 10 min at 8 °C. A 30 μL aliquot of the resulting supernatant was diluted into 970 μL of 50% (v/v) acetonitrile. The sample was then filtered through a 0.45 or 0.20 μm poly(vinylidene difluoride) (PVDF) filter into a high-performance liquid chromatography (HPLC) vial. Prior to each analysis a calibration curve of 0.1–100 μg/mL for ampicillin and 1–100 μg/mL for carbenicillin (in fresh LB media) was prepared using the above-mentioned method.

#### Detection and Quantification of Ampicillin and Carbenicillin by Liquid Chromatography–Tandem Mass Spectrometry (LC-MS/MS)

Analyses were performed using an ultraperformance liquid chromatography system (Waters) coupled to a Waters Xevo TQD Triple Quadrupole mass spectrometer with an electrospray ionization source. Quantitative analysis of the analytes was established by multiple reaction monitoring (MRM) in the positive mode. The tuning parameters, including collision energy, cone, and capillary voltages, were optimized by infusion of 1 mg/L solution of each analyte and IS in 50% (v/v) acetonitrile at a flow rate of 300 μL/min into the mass spectrometer.

LC-MS/MS analyses were performed by direct injection of 3 μL of sample (without a column) with 50% buffer A [2 mM ammonium acetate pH 4.7] and 50% buffer B [0.2% formic acid in acetonitrile] flowing at 0.3 mL/min. Nitrogen (650 L/h) and argon were used as the nebulizer and collision gases, respectively. Protonated molecular precursor [M + H]^+^ ions were identified at *m*/*z* 350.22 and 379.00 for ampicillin and carbenicillin, respectively. The most abundant ions corresponding to transitions ions 350 > 106 for ampicillin and 379 > 204 for carbenicillin were selected for quantification. An additional 4 for transitions for ampicillin and 3 for carbenicillin were monitored for verification when analyzing ampicillin. The transition of 350 > 114 was the only one used for verification of ampicillin (as the IS) when monitoring carbenicillin (the 3 transitions for carbenicillin remained unchanged). Optimum tuning parameters and the corresponding multiple reaction monitoring transitions are summarized in Table S3, SI. Mass Lynx software (version 4.1, Waters) was used for data processing and quantification was carried out manually.

#### LC-MS/MS Method Evaluation

Calibration curves were prepared prior to each analysis using fresh LB media spiked with different concentrations of the antibiotic of interest (see the Sample Work-Up section). Due to different sensitivities of each analyte, the linearities were obtained from 5 to 1000 ng/mL for ampicillin and 50–1000 ng/mL for carbenicillin with a coefficient of determination (*r*^2^) of 0.999. The limit of quantification (LOQ) for each analyte was considered as the lowest concentration of each calibration curve (S/N > 10).

Intraday quality controls (QC) were prepared as described in the Sample Work-Up section and measured at 5, 250, 400, and 500 ng/mL of ampicillin and 250, 400, and 500 ng/mL of carbenicillin in three replicates. The relative standard deviation (RSD) was calculated as 6–17% for ampicillin and 6–20% for carbenicillin (Table S4, SI).

#### Analyses of Potential Ion Suppression Effects of Internal Standards

Suppression of ampicillin signal by the IS was assessed by analyzing 1, 5, 10, 50, and 100 μg/mL ampicillin in LB in the presence and absence of 100 μg/mL carbenicillin. No significant suppression of the analyte was observed (Figure S4, SI). To assess the effects of spent media, curves were prepared in both fresh and spent LB media from BL21(*DE3*) using ampicillin and the IS. Briefly, cultures were grown for 4 h at 37 °C. Cells were removed by centrifugation at 3000*g* for 10 min, and calibration curves were prepared using 5, 10, 50, and 100 μg/mL of ampicillin. No significant difference between the curves was observed (Figure S4, SI).

#### Evaluation of Matrix Effect (ME) for the Detection and Quantification of Ampicillin and Carbenicillin in LB Media

To investigate the ME, the ratio of the peak area of the post extracted spiked LB media (*Ae*) to the peak area of standard solution (*Ab*) was calculated as the following equation
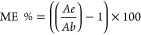
To measure the ME, three unspiked LB media samples were precipitated, diluted, and filtered as per the method described in the Sample Work-Up section. The filtrate was then evaporated under a stream of N_2_ gas and then reconstituted in aqueous solution containing 50, 100, and 150 ng/mL of the standards and the IS. ME was calculated by the equation above and was in the range of −15.2 to −31.5% for ampicillin and −33.3 to −41.5% for carbenicillin, indicative of suppression effects (Table S4, SI).

### Assessment of Plasmid Maintenance

A single colony of BL21(*DE3*) harboring *pET15b-sfgfp* (Tn3.1 and Tn3.1^MIN^) was grown as described in the Protein Expression section. After induction with 0.5 mM IPTG, a serial dilution was carried out and 100 μL of cells was plated out on LB agar, with and without ampicillin (20 μg/mL for Tn3.1^MIN^ and 100 μg/mL for Tn3.1). Colonies were counted using the OpenCFU program.^34^

### Selection in Back-Diluted Cultures

A single colony of BL21(*DE3*) harboring *pET28a-mcherry* (*aph*) was grown in LB media supplemented with 50 μg/mL kanamycin for 16–20 h at 37 °C with shaking. The following morning, the culture was back-diluted 1:100 into 10 mL of fresh media with 50 μg/mL kanamycin. This 10 mL of culture was spiked with 100 μL of either (1) fresh LB media, or (2) spent cell-free media from overnight cultures of BL21(*DE3*) harboring either *pET15b-sfgfp* (Tn3.1) or *pET15b-sfgfp* (Tn3.1^MIN^). This volume corresponded to a 1:100 dilution and was obtained by centrifuging 2 mL of overnight culture from *pET15b-sfgfp* (Tn3.1) and *pET15b-sfgfp* (Tn3.1^MIN^) at 13 000*g* for 1 min, then filtering the supernatant through a 0.2 μm filter (Whatman, England). The cultures were supplemented with either (1) no additional antibiotics, or (2) 20 ug/mL of ampicillin for Tn3.1^MIN^ and 100 ug/mL of ampicillin for Tn3.1. The growth of the BL21(*DE3*) harboring *pET28a-mcherry* was determined by measuring the OD_600_ in a 96-well SpectraMax *m2e* plate reader (Molecular Devices, U.K.).

### Alignment of Tn3 Fragments

Nucleotide sequence alignments using the nucleotide BLAST (nBLAST) service from National Center for Biotechnology Information were conducted on the Tn3.1 fragment from ref (39) and sequence of vectors mentioned in Table 1 from Addgene or Thermo Fisher. The Tn3.1 fragment^5^ was aligned to whole plasmid sequences to estimate the length of the Tn3 fragments. When investigating the *bla* gene sequences, only the *bla* genes were aligned, not the whole fragments. For identification of differences in the *P3* promoter region, the sequences from the 5′ end to the start codon of the *bla* gene were aligned.

## Supporting Information Available

Plasmids and primers used in the study, as well as optimization parameters for the mass spectrometry (PDF)
