# Supplementary material for: Antibiotic-Efficient Genetic Cassette for the TEM-1 β-Lactamase That Improves Plasmid Performance
Source: ACS Synth Biol. 2022 Jan 4;11(1):241–53. doi: 10.1021/acssynbio.1c00393 (PMC8787818; doi:10.1021/acssynbio.1c00393)
Supplement: Supplementary file 1 — sb1c00393_si_001.pdf [file sb1c00393_si_001.pdf]

Supporting Information

Antibiotic-Efficient Genetic Cassette for the TEM-1 ß-lactamase That Improves Plasmid Performance

Alister J. Cumming[1,#], Diana Khananisho[1,#], Ramona Harris[1], Carolyn N. Bayer[2], Morten H.H. Nørholm[2,3,4], Sara Jamshidi [5], Leopold L. Ilag[5] and Daniel O. Daley [1,3,4]

[1] Department of Biochemistry and Biophysics, Stockholm University, SE106 91, Sweden.

[2] The Novo Nordisk Foundation Center for Biosustainability, Technical University of Denmark, 2800, Denmark.

[3] CloneOpt AB, Upplands Väsby, SE194 68, Sweden.

[4] Mycropt ApS, Kongens Lyngby, 2800, Denmark.

[5] Department of Materials and Environmental Chemistry, Stockholm University, SE106 91, Sweden.

[#] Denotes that the authors contributed equally to the work

* Address correspondence to DOD (+46 8 162 910, ddaley@dbb.su.se)

Running Title: An antibiotic-efficient genetic cassette for the TEM-1 ß-lactamase

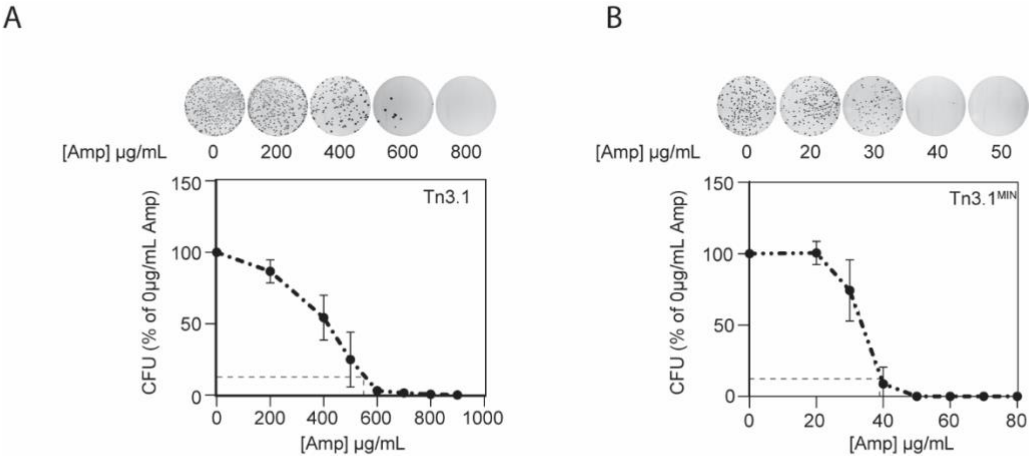

Figure S1. Excessive resistance conferred by Tn3.1 is mitigated by Tn3.1^{MIN} in BL21(*DE3*) pLysS. (A) BL21(*DE3*) pLysS harbouring the *pET15b-sfgfp* (Tn3.1) expression plasmid was plated on LB agar containing different concentrations of ampicillin. Colony numbers were normalised by the number of colonies that grew in the absence of ampicillin. The Minimum Inhibitory Concentration (MIC_{90}) of ampicillin required to kill 90 % of cells was extrapolated from the curve (dotted line) and deemed to be approximately 500 μg/mL. (B) As in panel (A) except that BL21(*DE3*) *pLysS* harbouring the *pET15b-sfgfp* (Tn3.1^{MIN}) plasmid were plated. The MIC_{90} was deemed to be approximately 38 μg/mL.

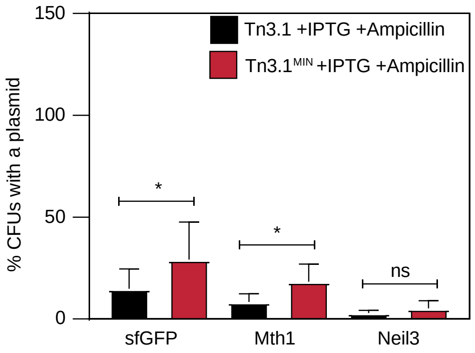

Figure S2. Tn3.1^{MIN} helps cells to maintain a plasmid. Upon induction of sfGFP, Mth1 or Neil3 production with IPTG, the majority of BL21(*DE3*) did not maintain a plasmid after 20 hours of induction (both Tn3.1 and Tn3.1^{MIN}). Data presented as mean ± s.d. ($n \geqslant 3$). A statistically significant difference of $p < 0.05$ (two-tailed Student's t-test) is denoted by *.

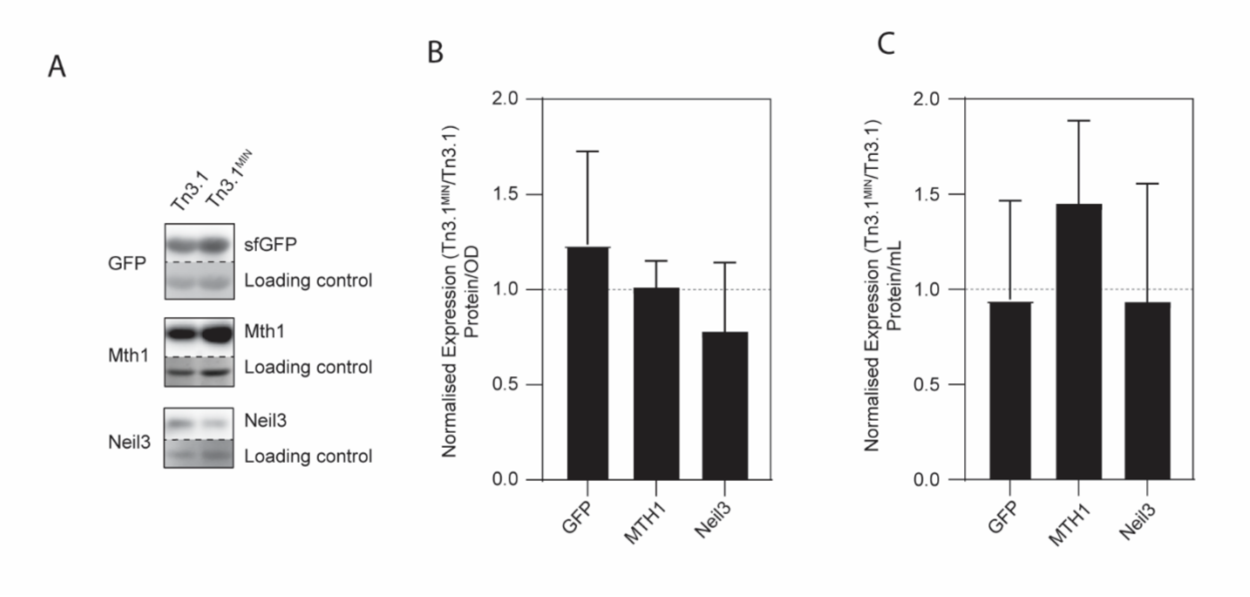

Figure S3. Tn3.1^{MIN} doesn't affect recombinant protein production. (A) GFP, Mth1 and Neil3 were expressed in BL21(*DE3*) using *pET15b* (Tn3.1 and Tn3.1^{MIN}). Cultures were induced with 0.5 mM IPTG for 20 hours, an OD_{600} of 0.05 was harvested and recombinant protein levels were assessed by SDS-PAGE and Western blotting using the HisProbe® HRP conjugate. Amido black or Ponceau staining was used for the loading controls. (B) Protein levels were quantified by densitometric analysis of the Western blots in panel A. Normalised expression levels were determined by calculating the ratio Tn3.1^{MIN} / Tn3.1. Values shown represent the relative amount of protein / OD_{600}. (C) As in B except that values represent the relative amount of protein/mL.

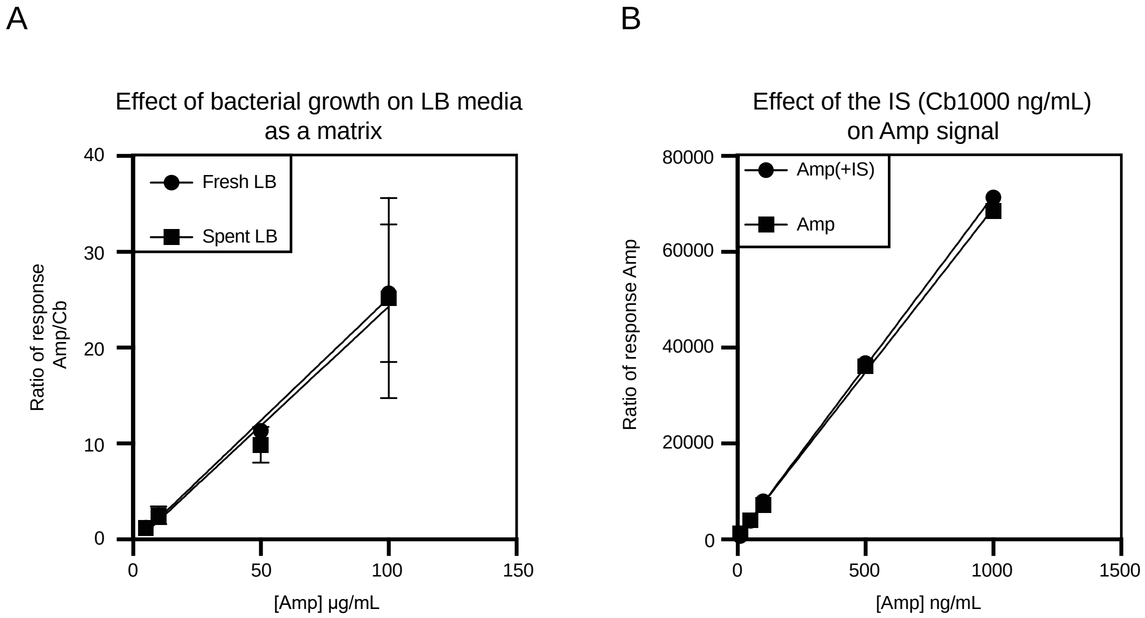

Figure S4. No significant effect from bacterial growth or IS on the ionisation of ampicillin. (A) Ampicillin was spiked at different concentrations into both fresh and spent (after 4 hours growth) LB media. Sample preparation was preformed including the addition of the IS and MS analysis performed by MS/MS. No significant difference was observed between the two slopes using a regression analysis. (B) Ampicillin was spiked at different concentrations into fresh LB media. Sample preparation was preformed including or omitting the addition of the IS and MS analysis preformed. No significant difference was observed between the two slopes generated in the presence or absence of the IS (using a regression analysis).

Table S1. Plasmids used in the study.

Plasmid	Origin of replication	Copy number	Ampicillin (µg/mL)	Carbenicillin (µg/mL)
pET15b-sfgfp (Tn3.1)	pBR322	Med	100	100
pET15b-sfgfp ($Tn3.1^{MIN}$)	pBR322	Med	20	20
pSEVA111	R6K	High	100	Nd
pSEVA121	RK2	Med	100	Nd
pSEVA131	pBBR1	Med	100	Nd
pSEVA141	pRO1600/ColE1	High	100	Nd
pSEVA151	RSF1010	Low/Med	100	Nd
pSEVA161	p15A	Med	100	Nd
pSEVA171	pSC101	Low	100	Nd
pSEVA181	pUC	High	100	Nd
pSEVA191	pBR322	Med	100	Nd
$pSEVA1^{MIN}11$	R6K	High	20	Nd
$pSEVA1^{MIN}21$	RK2	Med	20	Nd
$pSEVA1^{MIN}31$	pBBR1	Med	20	Nd
$pSEVA1^{MIN}41$	pRO1600/ColE1	High	20	Nd
$pSEVA1^{MIN}51$	RSF1010	Low/Med	<20	Nd
$pSEVA1^{MIN}61$	p15A	Med	20	Nd
$pSEVA1^{MIN}71$	pSC101	Low	<20	Nd
$pSEVA1^{MIN}81$	pUC	High	20	Nd
$pSEVA1^{MIN}91$	pBR322	Med	20	Nd
pET28a-mCherry	pBR322	Med	Na	Nd

Na denotes 'not applicable'
Nd denotes 'not determined'

Table S2. Primers used in the study.

Oligo Name	Sequence
AmpR TIR mut Fwd 1	CAATAATATTGAAAAAGGNNNNNNATGAGYATHCAACATTTCCGTGTCGCCC
AmpR TIR mut Fwd 2	CAATAATATTGAAAAAGGNNNNNNATGTCNATHCAACATTTCCGTGTCGCCC
AmpR TIR amp20 Fwd	CAATAATATTGAAAAAGGGGATGTATGAGTATTCAACATTTCCGTGTCGCCC
AmpR TIR mut Rev 1	CCTTTTTCAATATTATTGAAGCATTTATC
AmpR TIR Seq primer	CTTGAAGACGAAAGGGCC
pET15 CDS loop out Fwd	GGATCCGGCTGCTAACAAAGCCCGAAAGGAAGCTGAG
pET15 CDS loop out Fwd	GGGGAATTGTTATCCGCTCACAATTCCCCTATAGTGAGTCG
Insertion of MTH1 Fwd	AGCGGCCTGGTGCCGCGCGGCAGCCAT
Insertion of MTH1 Rev	CGGGCTTTGTTAGCAGCCGGATCCTTACACGGTATCAACTTCACGCAGGG
Insertion of Neil3 Fwd	AGCGGCCTGGTGCCGCGCGGCAGCCAT
Insertion of Neil3 Rev	GTTAGCAGCCGGATCCTTATTGACAGTGAGGACAGAAATATGTCATTCTGTTATTG
BlaCODOP loop out for pSEVA Fwd	CATTCAAATATGTATCCGCTCATGAGACAATAACC
BlaCODOP loop out for pSEVA Rev	TTACCAATGCTTAATCAGTGAGGCACCTATCTC
pSEVA loop out Fwd	CACTGATTAAGCATTGGTAAACCGATACAATTAAAGGCTCC
pSEVA loop out Rev	GGTTATTGTCTCATGAGCGGATACATATTTGAATG

Table S3. Optimum tuning parameters for the mass spectrometry of the analytes

Main working parameters for the mass spectrometry				
Capillary voltage (kV)	3.2			
Ion source temperature (° C)	150			
Desolvation temperature (° C)	350			
Analyte	Parent ion [M+H+]	Product ion	CE (V)	Cone Voltage (V)
Ampicillin	350	174	12	34
		106*	20	
		114	26	
		160	12	
		192	12	
Carbenicillin	379	204*	22	68
		220	16	
		160	16	
		114	42	

* Indicates the product ion used for quantification

Table S4. Parameters measured and values obtained for each analyte in the method evaluation

Analyte	LOQ[1] (ng/ml)	DLR[2] (ng/ml)	R^2	RSD%[3]				ME%[4]		
				5 (ng/mL)	250 (ng/mL)	400 (ng/mL)	500 (ng/mL)	50 (ng/mL)	100 (ng/mL)	150 (ng/mL)
**Ampicillin**	0.5	0.5-500	0.998	12	17	12	6	-15.2	-23.8	-31.5
**Carbenicillin**	5	5-500	0.996		20	6	18	-34.0	-41.5	-33.3

[1] denotes Limit of Quantification

[2] denotes Dynamic Linear Range

[3] denotes Relative Standard Deviation (precision)

[4] denotes Matrix Effect
